# Liquid alloy-driven ambient upcycling of fluoropolymer waste

**DOI:** 10.1093/nsr/nwag359

**Published:** 2026-06-12

**Authors:** Qingfeng Fu, Yan Duan, Yan Zhang, Jian Zhou, Peng Gao, Aiping Hu, Jilei Liu

**Affiliations:** College of Materials Science and Engineering, Hunan Joint International Laboratory of Advanced Materials and Technology for Clean Energy, Hunan Province Key Laboratory for Advanced Carbon Materials and Applied Technology, Hunan University, Changsha 410082, China; Research Institute of Hunan University in Chongqing, Chongqing 401120, China; College of Materials Science and Engineering, Hunan Joint International Laboratory of Advanced Materials and Technology for Clean Energy, Hunan Province Key Laboratory for Advanced Carbon Materials and Applied Technology, Hunan University, Changsha 410082, China; College of Materials Science and Engineering, Hunan Joint International Laboratory of Advanced Materials and Technology for Clean Energy, Hunan Province Key Laboratory for Advanced Carbon Materials and Applied Technology, Hunan University, Changsha 410082, China; College of Materials Science and Engineering, Hunan Joint International Laboratory of Advanced Materials and Technology for Clean Energy, Hunan Province Key Laboratory for Advanced Carbon Materials and Applied Technology, Hunan University, Changsha 410082, China; College of Materials Science and Engineering, Hunan Joint International Laboratory of Advanced Materials and Technology for Clean Energy, Hunan Province Key Laboratory for Advanced Carbon Materials and Applied Technology, Hunan University, Changsha 410082, China; College of Materials Science and Engineering, Hunan Joint International Laboratory of Advanced Materials and Technology for Clean Energy, Hunan Province Key Laboratory for Advanced Carbon Materials and Applied Technology, Hunan University, Changsha 410082, China; College of Materials Science and Engineering, Hunan Joint International Laboratory of Advanced Materials and Technology for Clean Energy, Hunan Province Key Laboratory for Advanced Carbon Materials and Applied Technology, Hunan University, Changsha 410082, China

**Keywords:** polytetrafluoroethylene, C–F bond, liquid alloy, chemical upcycling

## Abstract

The chemical upcycling of fluoropolymer waste, particularly polytetrafluoroethylene (PTFE), has been severely hampered by the harsh thermal conditions required to cleave the highly stable carbon–fluorine (C–F) bonds. Here, we report a contact-electro-catalysis-induced self-propagating reaction (CEC-SPR) strategy that enables efficient cleavage of the robust C–F bonds in PTFE under ambient conditions. By employing a highly reactive NaK alloy, which reacts with PTFE through the exothermic Wurtz defluorination reaction, we facilitate the chemical recycling of PTFE into fluorine-doped carbon materials and metal fluorides at room temperature. Our results provide a new conceptual strategy for the upcycling of PTFE plastic waste into functionally useful carbon materials and inorganic fluoride products.

## Teaser

A liquid-alloy-driven self-propagating strategy is proposed for fluoropolymer upcycling, in which robust C–F bonds are rapidly cleaved under ambient conditions to generate porous carbon materials and fluoride salts, providing a new route for fluoropolymer upcycling.

## INTRODUCTION

**Figure 1.**
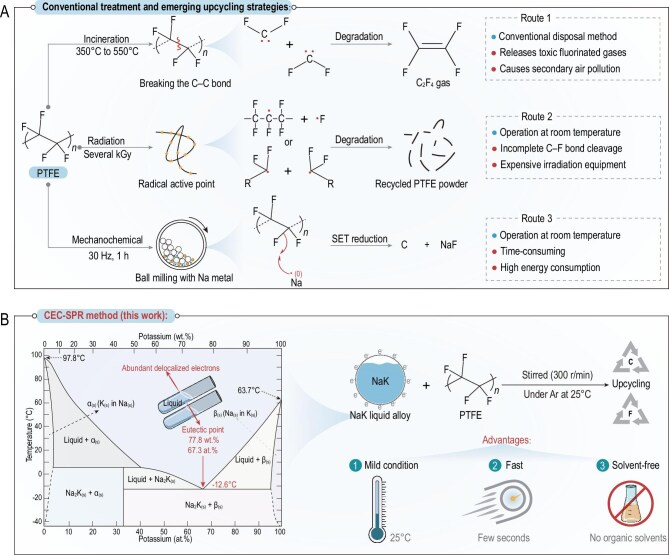
Recycling approach of PTFE. (A) Comparison of different conventional PTFE recycling methods. SET, single electron transfer. (B) Schematic illustration of the CEC-SPR upcycling method.

Plastic has become a fundamental material in modern society, playing a critical role in packaging, textiles, medical devices and transportation components. Despite its widespread use, <10% of plastic waste is currently recycled, with the majority either incinerated or landfilled, leading to significant environmental challenges such as water and air pollution [1–9]. Among various plastic types, polytetrafluoroethylene (PTFE), widely recognized as Teflon, is especially challenging [10–12]. Owing to its robust carbon–fluorine (C–F) bonds (485 kJ mol^−1^), PTFE exhibits outstanding chemical resistance, non-stick properties, and high thermal stability. While these structural characteristics make PTFE indispensable in industrial and consumer sectors, they also make it extremely resistant to biological and chemical degradation, resulting in significant and persistent environmental accumulation [13,14]. Currently, PTFE treatment routes are still largely dominated by thermal conversion (incineration and pyrolysis), which mainly generates fluorinated gaseous products, including C_2_F_4_, along with potentially hazardous species such as HF, particularly under uncontrolled or high-temperature conditions (Fig. 1A). To address these concerns, alternative approaches such as irradiation recycling have been explored. This method primarily targets the breaking of C–C bonds within the polymer backbone using high-energy gamma or electron irradiation (several kGy) [15–18]. Although irradiation recycling effectively reduces the molecular weight of PTFE, it typically results in low-grade products and significantly limits the subsequent applications of the material. Therefore, the development of novel, environmentally friendly upcycling strategies is urgently needed.

The direct conversion of inert C–F bonds in PTFE into valuable carbon and fluoride compounds is a highly appealing option but a challenging goal [19–23]. To achieve effective chemical degradation of PTFE, two key criteria must be met: (i) the presence of strong Lewis base sites and available electrons to interact with fluorine, thereby weakening the electron cloud of neighboring carbon atoms and facilitating C–F bond cleavage; and (ii) sufficient energy to sustain the reaction while preventing the degradation products from recombining to form C_2_F_4_ [20,24]. To alleviate these hurdles, Gouverneur and co-workers disclosed a phosphate/silicate-enabled mechanochemical protocol that degrades multiple classes of PFASs, while recovering fluoride for upcycling [20,25]. The approach highlights the improved C–F bond cleavage efficiency achieved through ball milling with inorganic salts such as K_3_PO_4_/K_4_P_2_O_7_ and Na_2_SiO_3_. Another interesting strategy involves the use of highly reducing alkali metals (for example, Li, Na, or K) to cleave the C–F bonds in PTFE through pyrolysis and high-energy milling [26–30]. However, these methods typically require extreme conditions such as high temperatures (∼350°C), high energy (ball milling) or extended reaction times (several hours) to release sufficient electrons to drive the reaction effectively. Despite these promising strategies, challenges remain in developing a degradation method for PTFE that can efficiently cleave stable C–F bonds under mild conditions while minimizing energy input.

Contact-electro-catalysis (CEC) is an emerging field that harnesses electron transfer at liquid–solid and liquid–liquid interfaces, driven by contact electrification, to initiate redox reactions. Its fundamental principle lies in utilizing external mechanical agitation and friction between dielectric materials (such as fluorinated ethylene propylene (FEP), poly(vinylidene fluoride) (PVDF), etc.) and liquids to induce contact electrification, which in turn generates interfacial electron transfer that drives catalytic chemical reactions [31,32]. Interestingly, PTFE is a promising negative triboelectric material with an ultralow dielectric constant (∼2.1), making it an ideal solid-state reaction medium for CEC [33]. In terms of the liquid phase, the Na_22_K_78_ alloy is a reactive liquid phase that offers abundant delocalized electrons and strong Lewis basicity (Fig. 1B). It remains liquid at room temperature due to its low eutectic point of −12.6°C, making it a promising working fluid for CEC [34,35]. Motivated by this, we developed a novel CEC strategy based on a NaK liquid alloy to rapidly degrade PTFE within seconds through mechanically induced self-propagating reactions (SPR). In this CEC-SPR reaction, PTFE is stirred in a liquid NaK alloy, where particle collisions break the C–F bonds, initiating exothermic reactions and generating a combustion wave (Supplementary Video). This combustion wave rapidly propagates through the system, forming self-propagating reactions that lead to the rapid degradation of PTFE. This work provides a new conceptual framework for fluoropolymer degradation and offers a new perspective for innovative recycling and upcycling strategies.

## RESULTS

**Figure 2.**
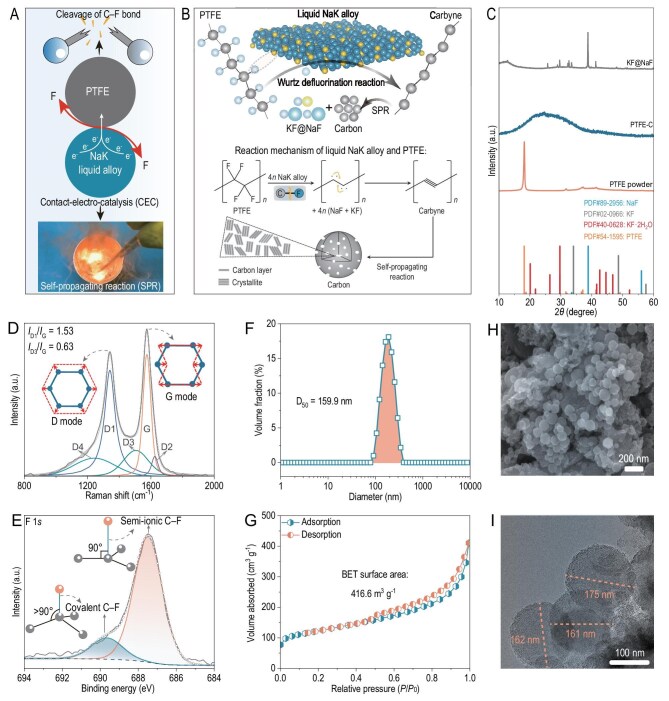
Characterization and defluorination mechanism of PTFE by liquid NaK alloy. (A) Schematic illustration of principle CEC-SPR reaction triggered by NaK alloy. (B) Proposed mechanism for PTFE defluorination involving electron transfer, alkali metal fluorides generation, and porous carbon material formation. (C) XRD patterns of PTFE, PTFE-C, and KF@NaF. (D) Raman spectrum of PTFE-C. (E) F 1*s* XPS spectrum of PTFE-C. (F) Particle size distribution of PTFE-C. (G) Nitrogen adsorption–desorption isotherms and BET surface area of PTFE-C. (H) SEM image and (I) TEM image of PTFE-C.

To elucidate the CEC-SPR reaction process, experiments were conducted in stainless steel batch reactors using commercial PTFE powder and NaK liquid alloy, as illustrated in Figs S1 and S2. In addition to providing excess electrons and Lewis base sites, NaK liquid alloy plays a critical role in initiating self-propagating reactions that drive the rapid degradation of PTFE. Its strong reducing capability facilitates nucleophilic attack on C–F bonds, contributing to the high degradation rate. Furthermore, owing to its low melting point (−12.6°C) and high boiling point (>700°C) [35,36], NaK liquid alloy can remain in the liquid phase throughout the reaction, ensuring intimate contact with PTFE, in contrast to solid pure Na or K metal chunks (Figs S3 and S4). The limited reactivity of solid Na or K under these conditions can be attributed to insufficient solid–solid interfacial contact and the absence of continuous electron transfer pathways, in contrast to the liquid NaK alloy. In addition, control experiments using Ga-based liquid metals showed no observable reaction with PTFE under similar conditions (Fig. S5), highlighting that the CEC-SPR behavior is not universal to all liquid metals but is closely associated with the strong reducing capability of NaK alloys. It is worth noting that in the absence of mechanical disturbance, no reaction occurs between PTFE and the NaK alloy (Fig. S6). Under stirring conditions, repeated frictional contact between PTFE particles and the liquid NaK alloy generates triboelectric charging at the dynamic interface, which promotes electron transfer from the electron-rich NaK alloy to PTFE (Fig. 2A). This interfacial electron injection perturbs the electron density around fluorine atoms, weakens the strong C–F bonds, and initiates the Wurtz-type defluorination reaction, in which released fluorine species are rapidly captured by Na/K to form alkali metal fluorides (Fig. 2B) [37]. The cleavage of the C–F bonds in PTFE is highly exothermic, and the released heat further accelerates bond dissociation and sustains the self-propagating reaction front. As a result, PTFE undergoes ultrafast defluorination within seconds at room temperature, yielding PTFE-derived amorphous carbon (PTFE-C), NaF, and KF, which can be subsequently separated via vacuum filtration (Fig. S7). Notably, similar self-propagating behavior was not observed for PVDF, PVC, or PE under comparable conditions (Fig. S8). This difference is likely associated with the fully fluorinated backbone of PTFE, whereas the presence of –CH_2_– segments in PVDF and PVC interrupts the propagation pathway.

Degradation products were characterized by X-ray diffraction (XRD). As shown in Fig. 2C, multiple diffraction peaks corresponding to KF, NaF, and KF·2H_2_O were clearly identified after centrifugal separation (Fig. S9), confirming the efficient defluorination of PTFE in the CEC-SPR reaction. Quantitative analysis further confirms the high defluorination efficiency. Elemental analysis indicates a total fluoride release of ∼91.1%, while ion chromatography reveals a fluoride yield of ∼89% corresponding to alkali metal fluorides (Table S1). In addition to these alkali metal fluorides, a broad diffraction feature was observed in the range of 20.0°–30.0°, corresponding to the (002) plane of amorphous carbon (denoted as PTFE-C). This result is in good agreement with the high-resolution transmission electron microscopy analysis (Fig. S10), indicating that the resulting PTFE-C has a relatively low degree of graphitization. To further investigate the structural disorder and chemical characteristics of PTFE-C, Raman analysis was therefore conducted. As shown in Fig. 2D, the Raman spectrum exhibits two dominant peaks at 1340 cm^−1^ (D4, D1, and D3 band) and 1576 cm^−1^ (G and D4 band), corresponding to the disordered carbon defects and the in-plane vibration of *sp*^2^ carbon atoms, respectively. The high intensity ratios of *I*_D1_/*I*_G_ (1.53) and *I*_D3_/*I*_G_ (0.63) indicate a high density of structural defects, probably introduced by F-doping during the fast defluorination process [38]. These abundant defect sites on the PTFE-C surface are crucial for modulating surface reactivity and offering abundant ion adsorption sites, thereby exerting a direct influence on its electrochemical performance.

To further analyze the functional groups in PTFE-C, X-ray photoelectron spectroscopy (XPS) analysis was carried out. As expected, the XPS spectrum confirms the presence of fluorine in the amorphous carbon matrix. The high-resolution F 1*s* spectrum shows two distinct peaks at 687.5 and 689.8 eV, attributed to semi-ionic C–F and covalent C–F bonds, respectively (Fig. 2E), indicating successful doping of F element into PTFE-C [38]. Combustion ion chromatography analysis indicates that ∼15.9% of the total fluorine is doped or retained in the carbon framework after the reaction. This result is further supported by energy-dispersive X-ray spectroscopy mapping, which reveals a uniform distribution of fluorine throughout the carbon framework (Fig. S11). The formation of C–F bonds in PTFE-C is intrinsically associated with the thermal decomposition of PTFE. During high-temperature pyrolysis, fluorine atoms are first detached from the polymer backbone, while part of the released fluorine species become incorporated into the emerging carbon matrix under the influence of rapid thermal shock. Due to the temporary nature of the re-doping process, semi-ionic C–F bonds are preferentially formed over covalent counterparts. These semi-ionic C–F bonds are known to facilitate ion transport and improve interfacial charge transfer kinetics, thereby improving the electrochemical performance of PTFE-C. Furthermore, fluorine doping can tailor surface chemistry by increasing surface acidity, which also enhances ion adsorption capacity.

The microstructures of PTFE-C were investigated by scanning electron microscopy (SEM) and transmission electron microscopy (TEM), which show a uniform nanosphere structure with an average particle size of ∼160 nm (Fig. 2F, H and I). Brunauer–Emmett–Teller (BET) further reveals a high specific surface area of 416.6 m^2^ g^−1^ and an average pore width of 3.8 nm (Fig. 2G and Fig. S12), indicating the highly porous structure of the nanospheres. The formation of nanospheres is attributed to the thermal stress generated during rapid pyrolysis [39], which is effectively dispersed by the spherical geometry, preventing structural fracture. In addition, the nanosphere structure reduces the surface energy, contributing to the thermodynamic stability of the amorphous carbon. Notably, the particle size and morphology of PTFE-C are largely independent of the initial PTFE particle size. As shown in Fig. S13, PTFE precursors with sizes ranging from ∼200 nm to ∼25 μm all yield carbon products with similar nanosphere structures (∼200 nm), indicating that the final morphology is governed by the rapid defluorination and structural reconstruction process rather than the precursor characteristics. To further evaluate the applicability to realistic feedstocks, commercial PTFE waste (for example, PTFE waste strips) were also tested (Fig. S14). Similar products, including amorphous carbon and alkali metal fluorides, were obtained, although the defluorination efficiency was slightly lower (∼88.9%), likely due to reduced interfacial contact compared to finely divided PTFE powder.

**Figure 3.**
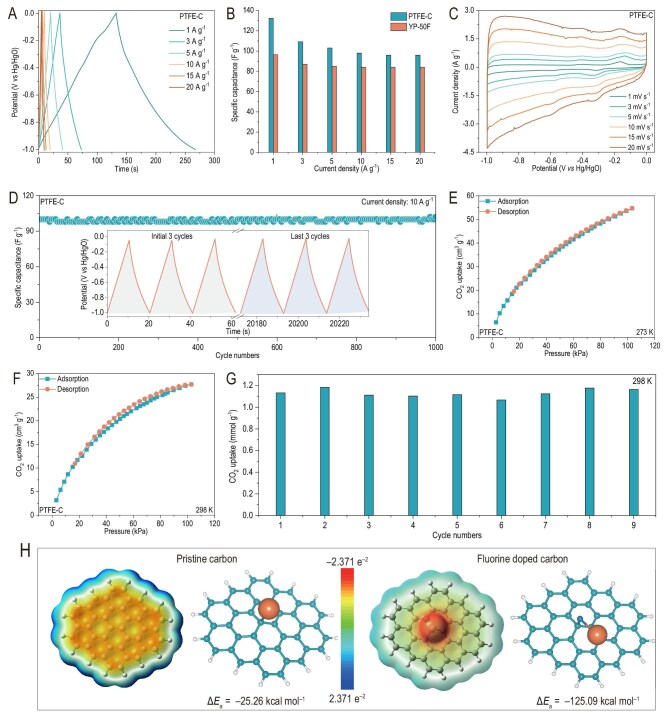
Evaluation of PTFE-C for supercapacitor and gas capture applications. (A) Galvanostatic charge/discharge curves at 1–20 A g^−1^ for PTFE-C. (B) Specific capacitance comparison between PTFE-C and commercial YP-50F at different current densities. (C) CV curves of the PTFE-C with different scan rates (1–20 mV s^−1^). (D) Cycling performance of PTFE-C at 10 A g^−1^ over 1000 cycles, with GCD profiles of the initial and final three cycles (inset). (E and F) CO_2_ adsorption and desorption isotherms of PTFE-C at 273 and 298 K. (G) CO_2_ adsorption and desorption cycle test for PTFE-C at 298 K. (H) DFT computations of K^+^ adsorption energies of pristine carbon and fluorine-doped carbon, respectively.

Given the favorable structural features, including high specific surface area, uniform nanosphere morphology, and abundant fluorine-containing functional groups, the electrochemical performance of PTFE-C was systematically evaluated in a symmetric supercapacitor configuration. As shown in Fig. 3A, PTFE-C exhibits a high specific capacitance of 132.4 F g^−1^ at a current density of 1 A g^−1^, and maintains an impressive capacitance of 96.0 F g^−1^ even at 20 A g^−1^, demonstrating excellent rate capability. In contrast, commercial YP50F exhibits lower capacitance values of 96.4 and 84.0 F g^−1^ at the corresponding current densities (Fig. 3B and Fig. S15). The cyclic voltammetry curves in Fig. 3C show a quasi-rectangular shape at different scan rates, further confirming the excellent capacitive characteristics of PTFE-C. To evaluate its cycling stability, a long-term stability test was conducted at 10 A g^−1^. As expected, the PTFE-C electrode exhibits outstanding capacitance retention over 1000 cycles (Fig. 3D), demonstrating excellent cycling stability. The inset of Fig. 3D shows overlapping charge-discharge curves for the initial and final cycles, indicating negligible degradation in electrochemical performance. To further evaluate the stability of fluorine species during electrochemical operation, ion-selective electrode (ISE) measurements were conducted on the electrolyte after cycling. The detected fluorine concentration was as low as ∼0.033 mg L^−1^, indicating negligible fluorine leaching and confirming the strong binding of fluorine within the carbon framework. The enhanced electrochemical properties of PTFE-C are due to fluorine doping, which modulates its electronic structure and optimizes charge storage characteristics, making it significantly superior to conventional activated carbons such as YP50F. The proposed mechanism is further supported by density functional theory (DFT) calculations (Fig. 3H). Specifically, the calculated K^+^ adsorption energy (∆*E*_a_) on PTFE-C (−125.09 kcal mol^−1^) is significantly lower than that on pristine carbon sheets (−25.26 kcal mol^−1^), indicating stronger K^+^ binding and improved ion transfer charge diffusion, which directly contributes to the superior performance of PTFE-C in supercapacitor applications. In addition to energy storage applications, the porous structure of PTFE-C was also explored by CO_2_ adsorption. The adsorption–desorption isotherms at 273 and 298 K (Fig. 3E and F) indicate a substantial CO_2_ uptake, suggesting its potential for carbon capture applications. Moreover, as illustrated in Fig. 3G, the CO_2_ adsorption capacity remains stable over 9 cycles, indicating good cycle stability and adsorption efficiency. Overall, these findings demonstrate that amorphous carbon derived from PTFE degradation not only exhibits excellent supercapacitor performance but also holds promise for CO_2_ adsorption applications, providing a dual-functional material with significant environmental and energy-related benefits.

**Figure 4.**
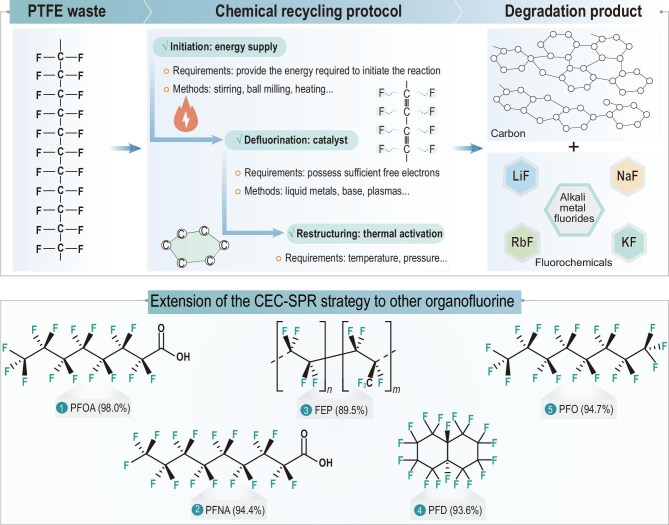
Schematic illustration of a universal strategy for chemical recycling of PTFE and other organofluorine compounds.

Inspired by the CEC-SPR reaction developed here for PTFE upcycling using a liquid NaK alloy, we further propose a generalized chemical recycling framework for fluoropolymers, with PTFE taken as a representative example (Fig. 4). This framework comprises three interdependent stages: initiation, defluorination, and structural reconstruction. In the initiation stage, an external energy input is required to activate the inert fluoropolymer surface and trigger interfacial reactions. In the present work, this role is fulfilled by mechanical agitation, whereas ball milling, heating, plasma activation, or other stimuli may serve similar functions in related systems. In the defluorination stage, electron-rich catalysts or reactive media (for example, liquid metals, inorganic bases, or plasmas) promote C–F bond cleavage and release fluorine species. In our CEC-SPR system, the liquid NaK alloy acts as both the electron donor and fluorine scavenger. In the structural reconstruction stage, the liberated fluorine species combine with alkali metals to form stable fluorides (for example, LiF, NaF, KF, and RbF), while the carbon backbone undergoes rapid reorganization into disordered or graphitic carbon frameworks. In this work, this process leads to the formation of PTFE-derived carbon (PTFE-C). This generalized framework captures the common physicochemical principles underlying fluoropolymer conversion and may provide guidance for the future development of sustainable fluoropolymer recycling technologies. While the present work primarily establishes a conceptual framework, further efforts in reaction engineering, cost optimization, and safety control will be required to advance toward scalable and practical implementation.

Furthermore, the proposed strategy can also be applied to various organofluorine compounds, such as perfluorooctanoic acid (PFOA, Table S2 and Fig. S16), perfluorononanoic acid (PFNA, Table S3 and Fig. S17), perfluoroethylene propylene copolymer (FEP, Table S4 and Fig. S18), perfluorodecahydronaphthalene (PFD, Table S9 and Fig. S19), and perfluoro-n-octane (PFO, Table S10 and Fig. S20), highlighting its broad applicability and potential for generalized fluorochemical recycling (Note: Avoid use with energetic polymers and fluorinated amines).

## DISCUSSION

In summary, we present a novel and complementary strategy for chemically degrading PTFE via a CEC-SPR induced by a NaK liquid alloy. This approach enables rapid and energy-efficient cleavage of the inert C–F bonds under ambient conditions, yielding valuable products including amorphous carbon and alkali metal fluorides. The resulting PTFE-C exhibits a nanosphere morphology, high surface area, and partial fluorine doping, which significantly enhances its electronic structure. As a supercapacitor electrode, PTFE-C demonstrates excellent specific capacitance, rate capability, and long-term cycling stability. Additionally, its porous structure facilitates substantial CO_2_ adsorption with good recyclability. This work not only provides a conceptual strategy for PTFE waste recycling but also offers new insights into the activation of inert C–F bonds in fluorinated polymers.

## Supplementary Material

nwag359_Supplemental_Files
