# Supplementary material for: Liquid alloy-driven ambient upcycling of fluoropolymer waste
Source: Natl Sci Rev. 2026 Jun 12;13(14):nwag359. doi: 10.1093/nsr/nwag359 (PMC13397524; doi:10.1093/nsr/nwag359)
Supplement: nwag359_Supplemental_Files [file nwag359_supplemental_files.zip › Supplementary data.pdf]

Supplementary Materials for

Liquid alloy driven ambient upcycling of fluoropolymer waste

Qingfeng Fu, Yan Duan, Yan Zhang, Jian Zhou, Peng Gao, Aiping Hu, Jilei Liu*

Corresponding author. E-mail: liujilei@hnu.edu.cn

The PDF file includes:

Materials and Methods

Safe Handling Protocols for NaK Liquid Alloy in PTFE Upcycling Experiments

Supplementary Notes 1 to 12

Figs. S1 to S20

Tables. S1 to S6

Other supporting material for this manuscript include the following:

Movie S1

Materials and Methods

Materials synthesis

Polytetrafluoroethylene (PTFE) powder (provided by Energy Chemical) was used as the fluoropolymer precursor without further purification. The NaK alloy was freshly prepared by mixing metallic sodium and potassium chunk in a molar ratio of 22:78 under an inert argon atmosphere inside a glovebox (Please see "**Safe Handling Protocols for NaK Liquid Alloy in PTFE Upcycling Experiments**" Section for technical details). In a typical recycling reaction, a mixture of PTFE (200 nm, 0.738g) and NaK alloy (1g) was vigorously stirred in a sealed stainless-steel reactor under an argon atmosphere (**Figure S1**). The reactor was then mechanically stimulated to initiate a contact-electro-catalysis induced self-propagating reaction (CEC-SPR). After cooling to room temperature, the solid residue was collected, washed with deionized water several times, and dried under vacuum at 120°C for 12 h.

Note: Avoid use of NaK alloy with energetic polymers and fluorinated amines.

Electrochemical measurements

The electrochemical performance of recycled PTFE-C was evaluated in a three-electrode system using 6 M KOH aqueous solution as the electrolyte. The working electrode was prepared by mixing PTFE-C (80 wt%), acetylene black (10 wt%) and polyvinylidene fluoride (PVDF) binder (10 wt%) in N-methylpyrrolidinone (NMP) to form a homogeneous slurry. The resulting slurry was uniformly coated onto a nickel foam substrate, dried at 80°C under vacuum for 12 h. Pt electrode and Hg/HgO electrode were used as counter electrode and reference electrode, respectively. Cyclic voltammetry (CV) and galvanostatic charge-discharge (GCD)were performed using a Solartron Analytical 1470E electrochemical workstation.

Materials characterization

The phase composition of the degradation products was analyzed using X-ray diffraction (XRD, Bruker D8 Advance) with Cu Kα radiation. The morphology and microstructure were observed by field-emission scanning electron microscopy (SEM, Hitachi S-4800, Japan) and transmission electron microscopy (TEM, Thermo Fisher Scientific microscope Themis Z 3.2). Surface chemical states were investigated using X-ray photoelectron spectroscopy (XPS, Shimadzu Co, AXIS SUPRA). Raman spectroscopy (WITEC alpha-300R) was applied to determine the graphitization degree of carbon residues. Brunauer-Emmett-Teller (BET) specific surface area (SSA) was determined using nitrogen adsorption-desorption isotherms (JW-BK200C, Beijing JWGB SCI & TECH). Elemental analysis (C, H, O, and N) was performed using an Elementar UNICUBE analyzer, and fluorine content was determined using a combustion ion chromatography system (CIC-3200/HIC).

Computations

All calculations were carried out using the Gaussian 16 software package. The B3LYP functional in combination with the 6-31G(d) basis set was employed for geometry optimizations and energy calculations. Frequency analyses were performed to confirm the nature of the stationary points; no imaginary frequencies were found for the optimized minima, indicating that the structures correspond to true local minima on the potential energy surface. To better understand the interaction between fluorine species and carbon substrates, electrostatic potential (ESP) maps were generated based on the optimized structures. Visualization of the molecular electrostatic potentials was conducted using GaussView software, both for pristine carbon structures and those after fluorine attachment.

Safe Handling Protocols for NaK Liquid Alloy in PTFE Upcycling Experiments

Hazard Overview:

NaK (sodium-potassium) alloy is a highly reactive liquid metal alloy at room temperature (eutectic point: −12.6 °C). It reacts violently with water, moisture, and many common solvents, releasing hydrogen gas and substantial heat, which may result in fire or explosion. The alloy also causes severe chemical burn hazards upon skin contact and may generate corrosive fumes (*e.g.*, NaOH/KOH aerosols) when exposed to air or humidity.

Personal Protective Equipment (PPE):

All personnel must wear full PPE, including chemical safety goggles with a face shield, heavy-duty impervious gloves, a fire-retardant lab coat or apron, and closed-toe (preferably leather) shoes.

Engineering Controls:

All operations involving NaK alloy must be conducted either in an argon-filled glovebox or in a well-ventilated fume hood specifically certified for pyrophoric and highly reactive materials. Oxygen and moisture levels should be maintained below 0.01 ppm to prevent ignition or uncontrolled reactions. Only sealed, dry, and chemically compatible equipment (*e.g.*, stainless-steel reactors and stainless-steel stir bars) should be used, as described in the manuscript.

Handling and Operational Precautions:

All operations involving NaK alloy were conducted under strictly inert conditions. The NaK alloy (Na:K = 22:78) was prepared in an inert-atmosphere glovebox, handled using pre-dried tools, and charged together with PTFE into a sealed, pressure-rated stainless-steel reactor under argon. Mechanical agitation was performed using leak-free equipment, and rigorous moisture exclusion was maintained by thoroughly drying all reagents, solvents, and vessels prior to use.

Fire Safety Measures:

A Class D fire extinguisher (suitable for metal fires) and a container of dry sand or dry graphite powder must be readily accessible in the working area. Water, CO_2, or standard ABC fire extinguishers must not be used for NaK-related fires. In the event of a small spill or ignition, smother the fire with dry sand or use a Class D extinguisher. If the fire cannot be controlled, evacuate immediately and contact emergency services.

Supplementary Note 1: *Information about the reaction vessel.*

All reactions involving NaK alloy were performed in sealed stainless-steel reactors. All components in contact with NaK were made exclusively of stainless steel to ensure chemical compatibility and safe operation under highly reactive conditions (**Figs S1** and **S2**).

All experiments were performed in an argon-filled glovebox.

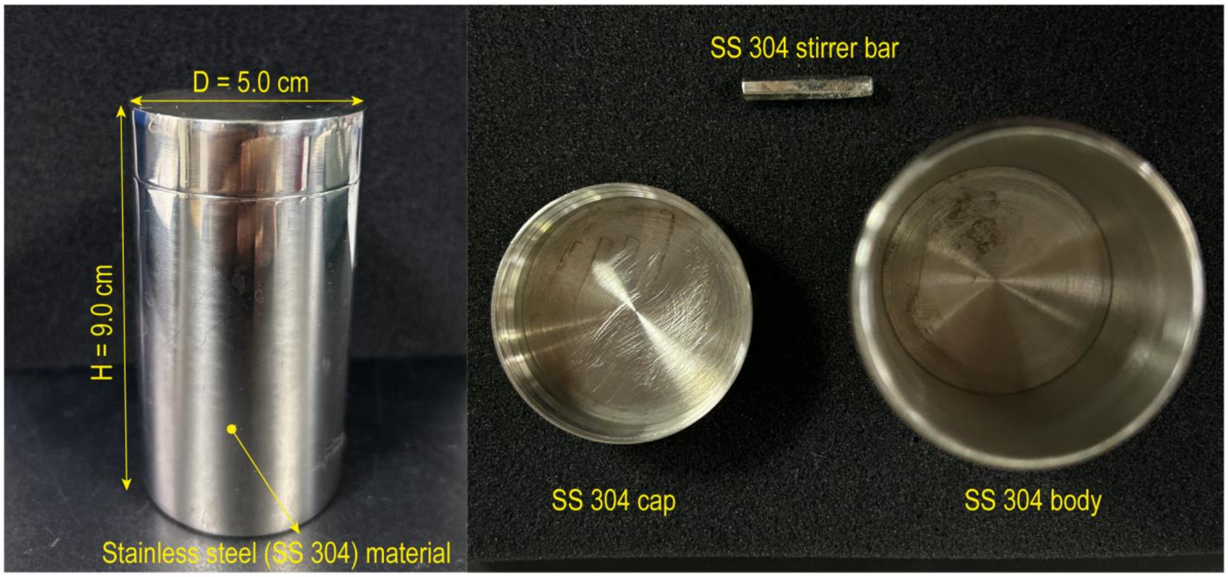

Fig. S1. Schematic diagram of stainless-steel reactor.

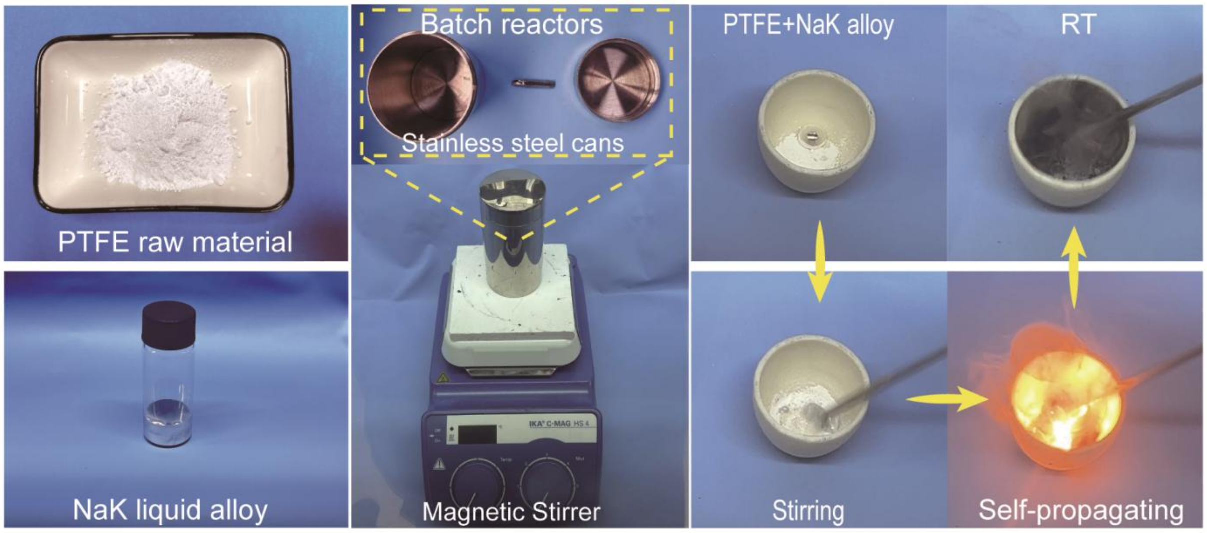

Fig. S2. The setup of CEC-SPR reaction.

Supplementary Note 2: *Influence of Alkali Metal State on PTFE Defluorination.*

Pure Na (melting point: 97.8°C) or K (melting point: 63.2°C) chunks is solid at room temperature and requires melting or high-energy ball milling to react (**Fig. S3**), which leads to limited interfacial contact and inefficient electron transfer, resulting in sluggish and non-uniform reactions (**Fig. S4**). These results further highlight the unique advantage of the NaK-alloy system for PTFE degradation under ambient conditions.

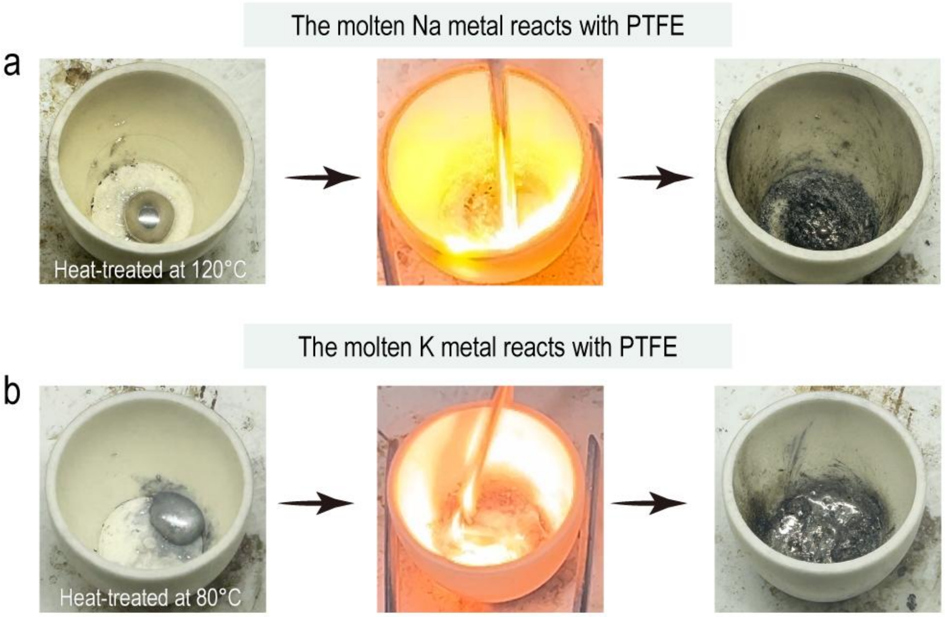

Fig. S3. The molten (a) Na metal and (b) K metal reacts with PTFE.

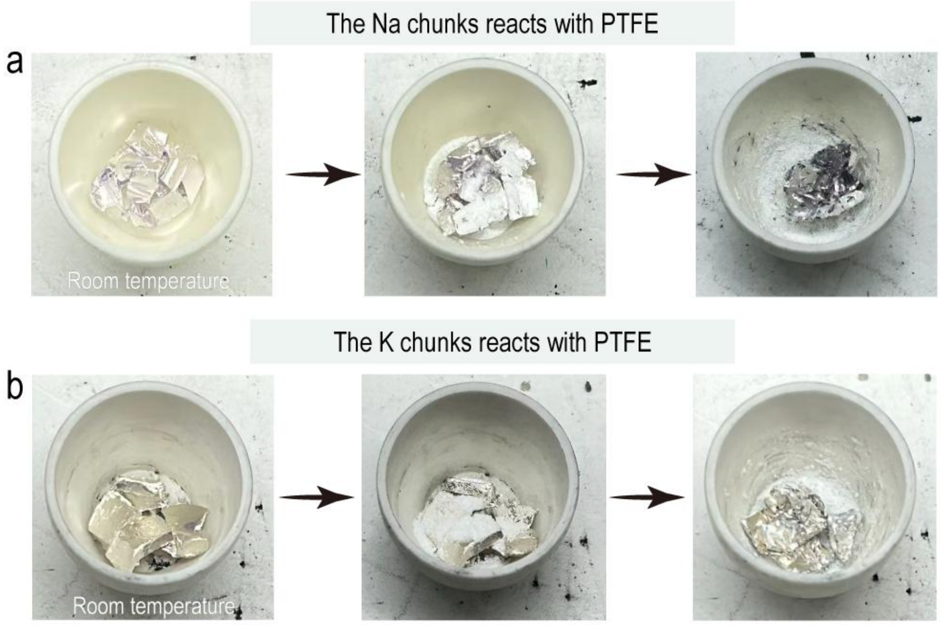

Fig. S4. (a) Na metal chunks and (b) K metal chunks show no observable reaction with PTFE under conventional stirring conditions.

Supplementary Note 3: *Rationale for the Selection of NaK Alloy.*

The selection of NaK alloy in this study was based on its unique combination of physicochemical properties. First, NaK possesses exceptionally strong reducing ability originating from alkali metals, which is thermodynamically favorable for the cleavage of robust C–F bonds. Second, the eutectic alloy exhibits a low melting point (–12.6°C), allowing it to remain liquid at room temperature and maintain continuous interfacial contact with PTFE. Third, the liquid-state interface can be continuously renewed under mechanical stimulation, which facilitates interfacial electron transfer during the reaction process. In contrast, Ga-based liquid metals, although fluid under near-ambient conditions, possess significantly weaker reducing ability and showed no observable reaction toward PTFE under comparable conditions (**Fig. S5**). These observations indicate that the CEC-SPR behavior is not universal to all liquid metals, but is closely related to the strong reducing nature and liquid-state interfacial characteristics of NaK alloys.

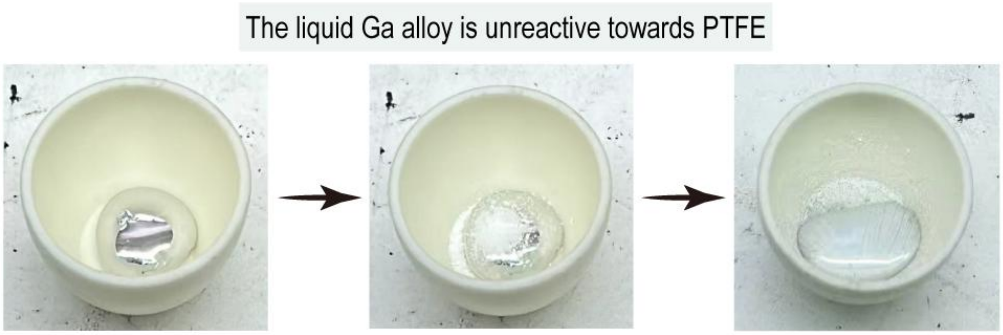

Fig. S5. The liquid Ga metal shows no observable reaction with PTFE.

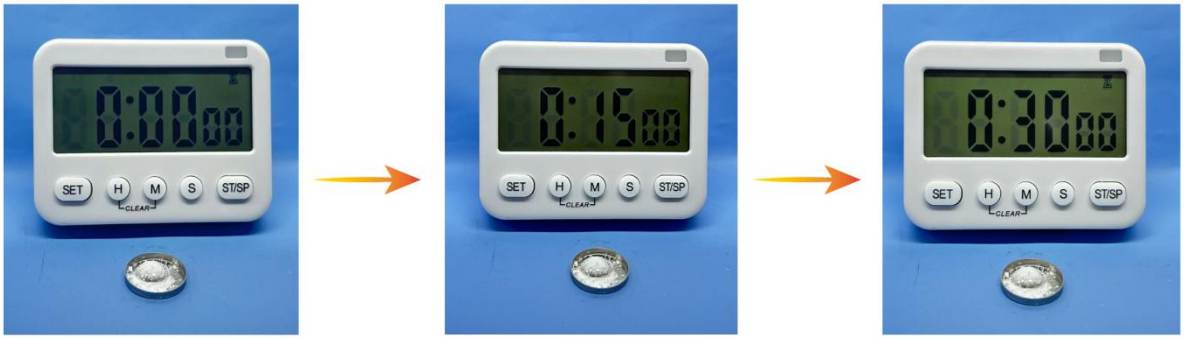

Fig. S6. No reaction is observed between PTFE and the NaK alloy without mechanical disturbance.

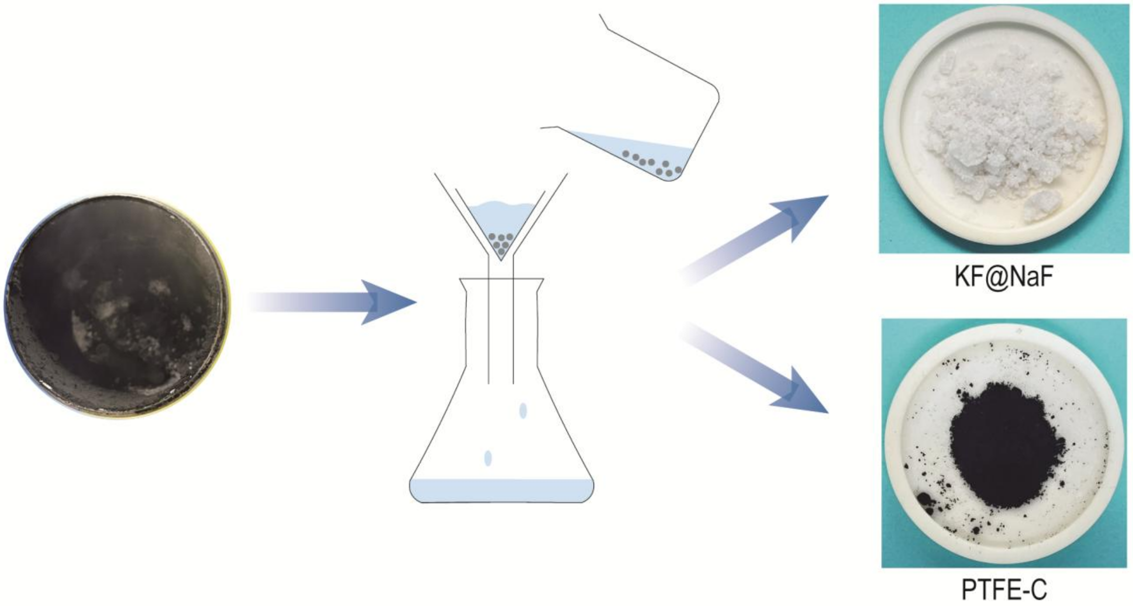

Fig. S7. Schematic illustration of product separation via vacuum filtration.

Supplementary Note 4: *Reactivity of different polymers toward NaK alloy.*

To evaluate the generality of the present strategy, we additionally examined several representative polymers, including PVDF, PVC, and PE, under similar conditions. Interestingly, no self-propagating reaction behavior was observed for these materials. We speculate that the unique reactivity of PTFE originates from its fully fluorinated backbone, which enables continuous C–F bond cleavage and propagation of the exothermic defluorination process. In contrast, the presence of $–CH_2–$ segments in PVDF and PVC interrupts the continuous fluorinated structure and hinders the rapid propagation of C–F bond cleavage (**Fig. S8**). Non-halogenated polymers such as PE lack reducible C–X bonds and may require alternative activation pathways. These results suggest that the CEC-SPR behavior is closely associated with the fully fluorinated PTFE structure rather than being universally applicable to all polymer wastes.

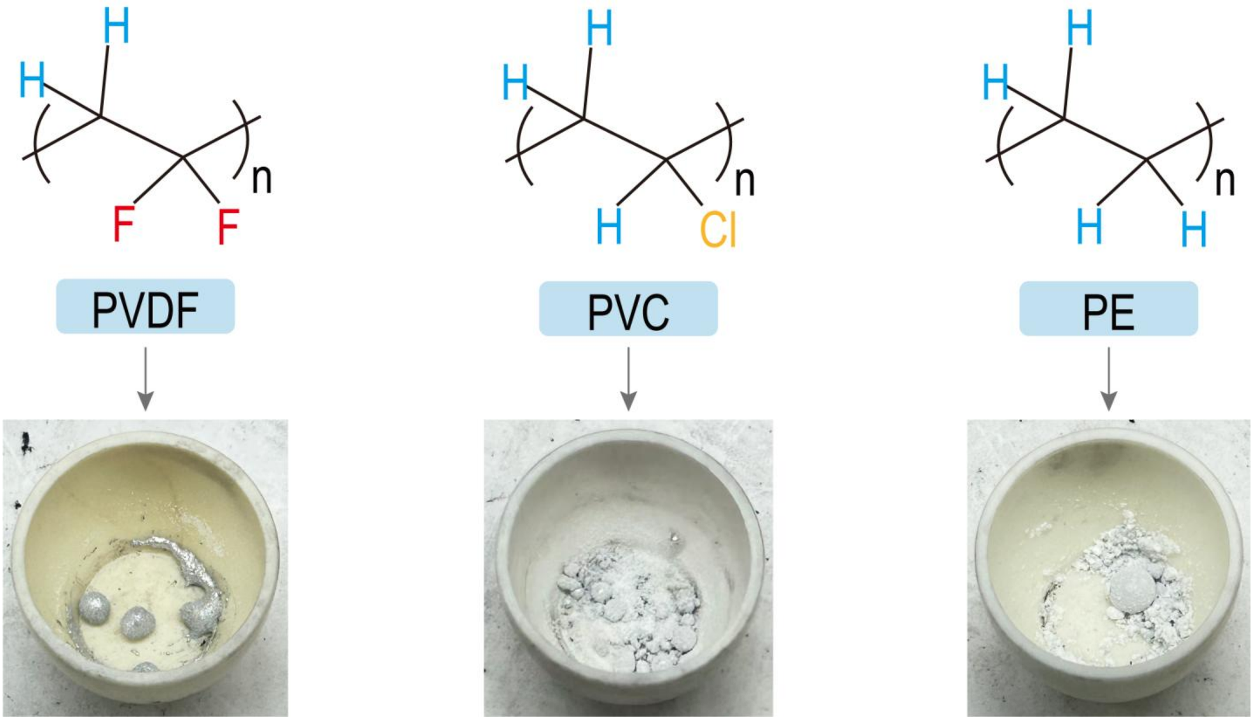

Fig. S8. Extension of the CEC-MSR strategy for other polymer compounds.

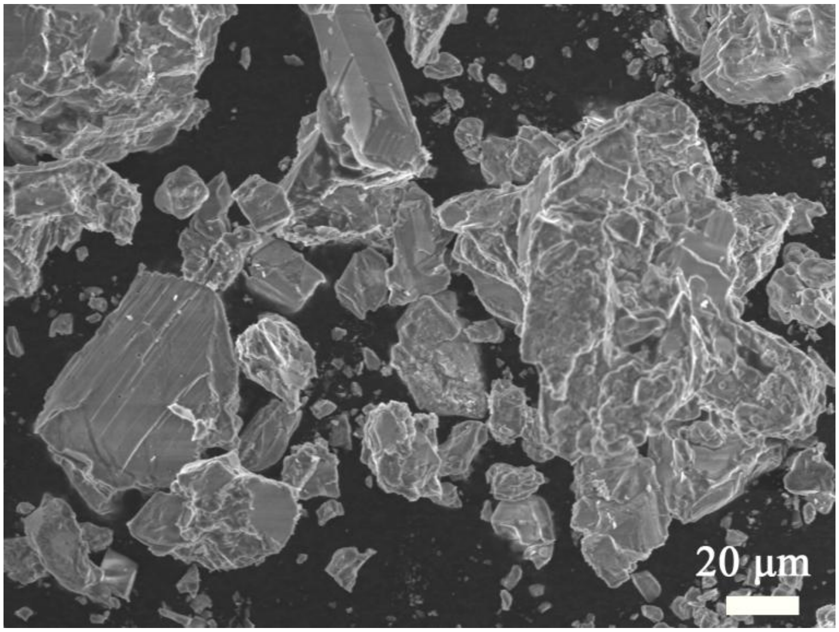

Fig. S9. SEM of KF@NaF.

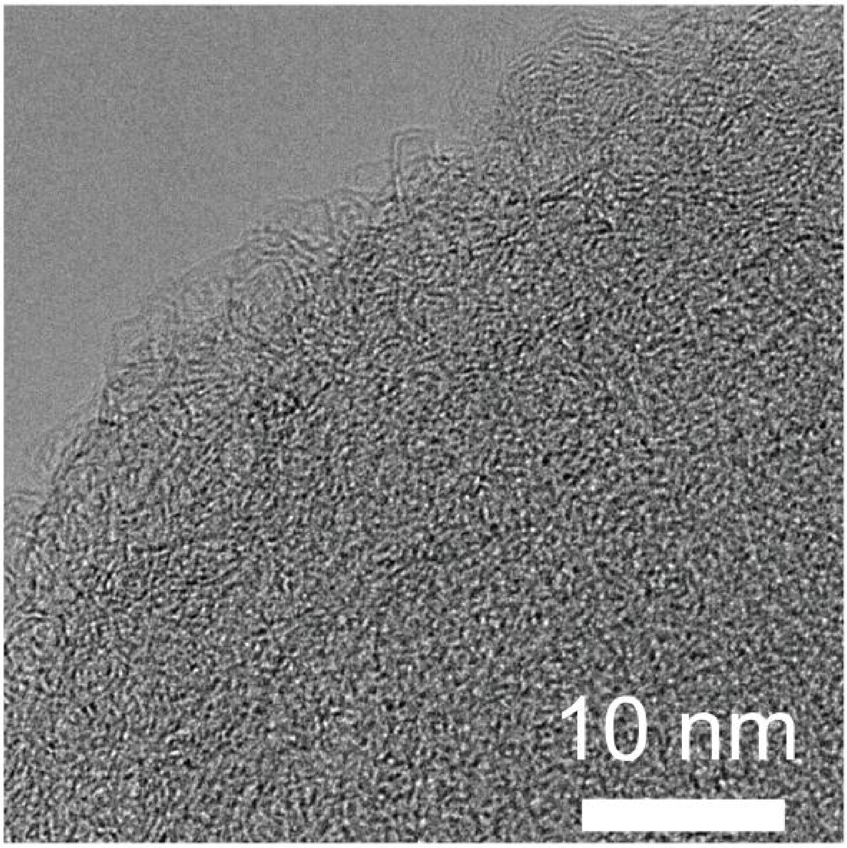

Fig. S10. HRTEM of PTFE-C.

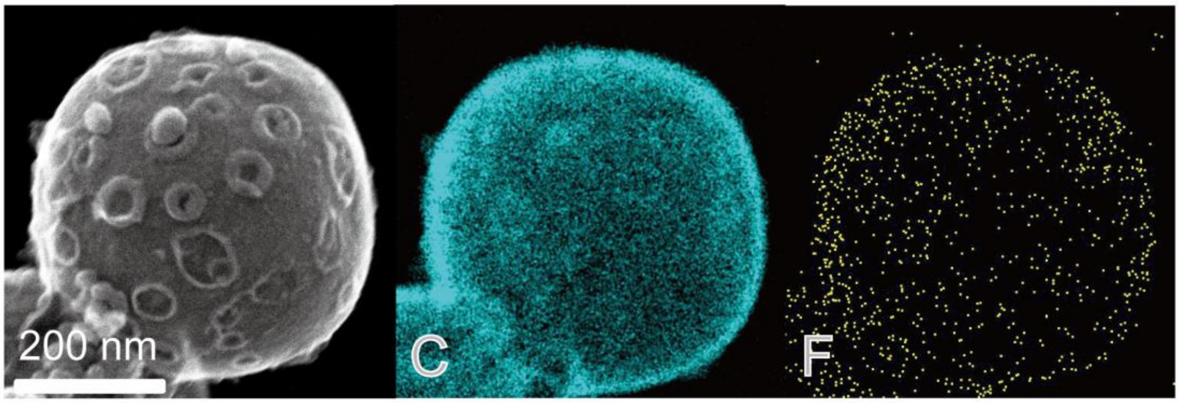

Fig. S11. EDS mapping of PTFE-C.

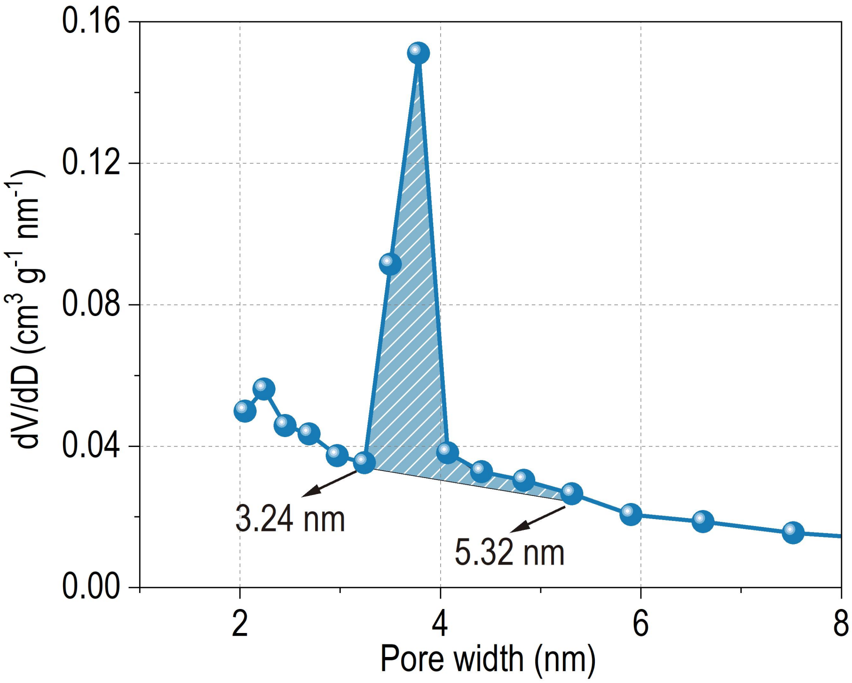

Fig. S12. Pore size distributions of PTFE-C.

Supplementary Note 5: *Influence of initial PTFE characteristics on the morphology of PTFE-C.*

To evaluate the influence of precursor characteristics on the morphology of the resulting carbon products, PTFE samples with different initial particle sizes were subjected to the CEC-SPR reaction under identical conditions. The tested PTFE precursors covered a broad size range from approximately 200 nm to 25 μm. The results show that the particle size and morphology of the obtained PTFE-derived carbon (PTFE-C) are largely independent of the initial PTFE particle size. Regardless of the precursor morphology, the resulting products consistently exhibit nanosphere structures with particle sizes in the range of approximately 160–200 nm (**Fig. S13**). These observations indicate that the final morphology of PTFE-C is primarily governed by the rapid defluorination process and subsequent structural reconstruction during the CEC-SPR reaction, rather than by the initial particle size or molecular weight of the PTFE precursor.

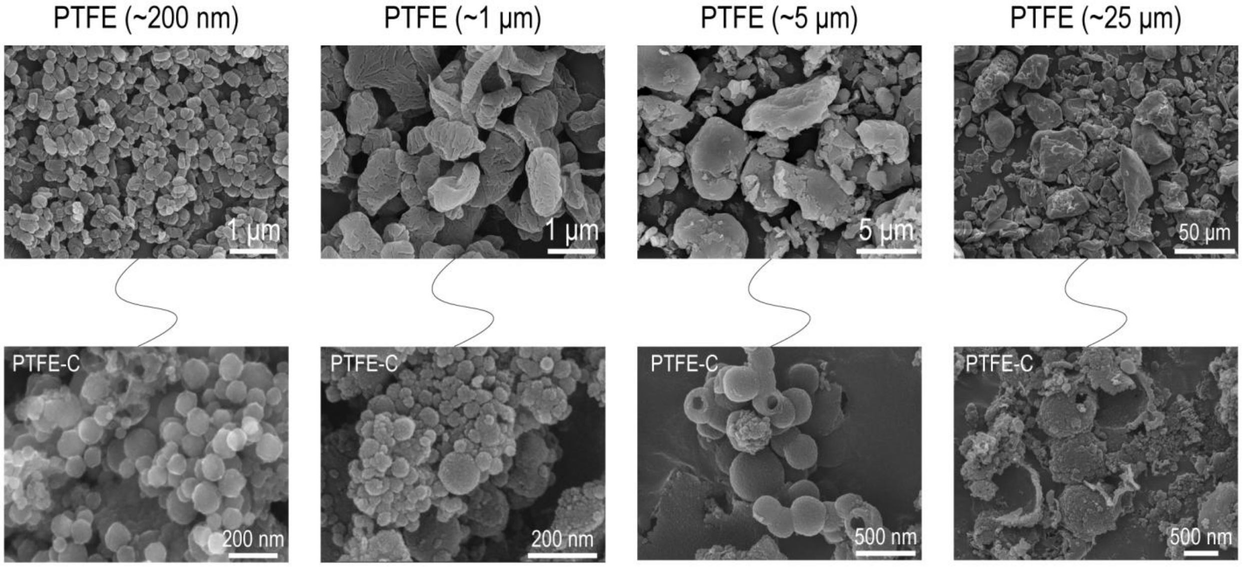

Fig. S13. SEM images of PTFE powder with different particle size and corresponding PTFE-C.

Supplementary Note 6: *Applicability of the CEC-SPR strategy to commercial PTFE waste.*

To evaluate the applicability of the CEC-SPR strategy to realistic fluoropolymer feedstocks, additional experiments were conducted using commercial PTFE waste (*e.g.*, PTFE waste strips) instead of reagent-grade PTFE powder. The commercial PTFE samples were cut into small pieces and subjected to the same reaction conditions as those used for PTFE powder. The results demonstrate that the CEC-SPR process can also be applied to non-powder PTFE feedstocks. Similar products, including PTFE-derived amorphous carbon and alkali metal fluorides, were obtained after reaction (**Fig. S14**). The defluorination efficiency was slightly lower than that of finely divided PTFE powder (~88.9%), which is attributed to the reduced interfacial contact area between the solid PTFE substrate and the liquid NaK alloy. These observations further highlight the critical role of interfacial contact in the CEC-SPR process and suggest that pretreatment of practical PTFE waste (*e.g.*, cutting, grinding, or size reduction) may improve reaction efficiency. The present results demonstrate the feasibility of extending this strategy to realisticPTFE waste streams.

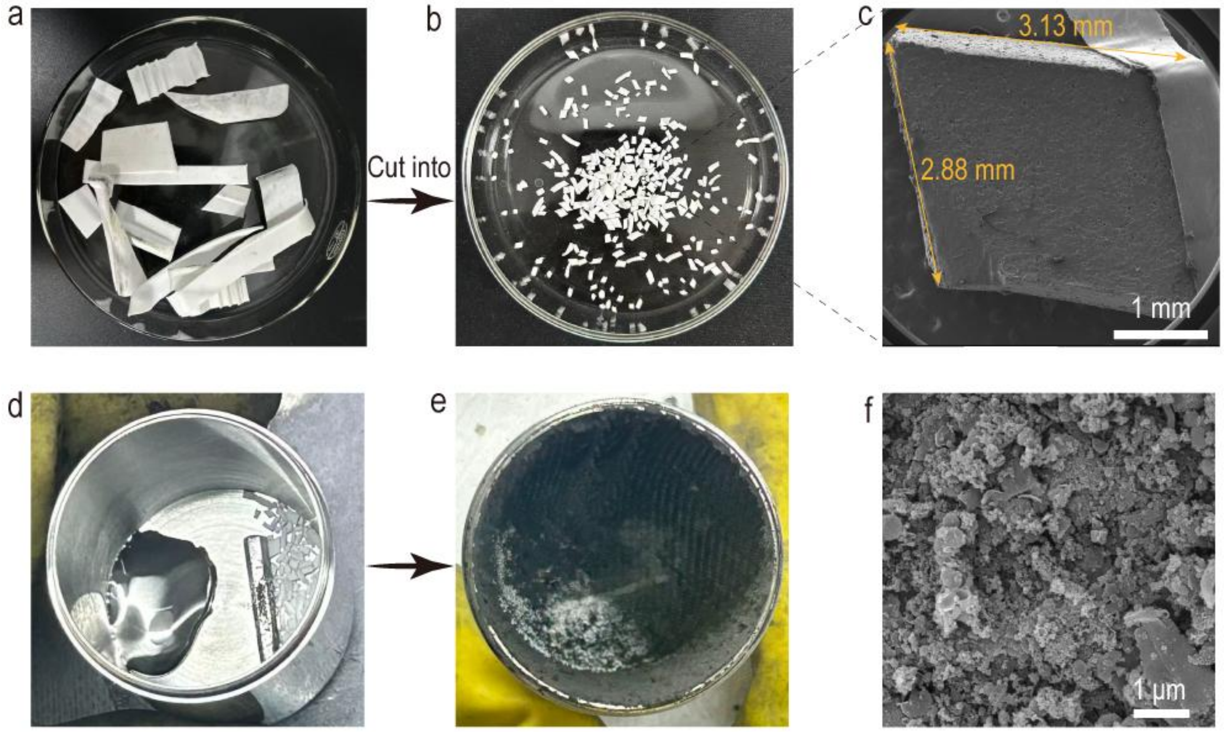

Fig. S14. Applicability of the CEC-SPR strategy to commercial PTFE waste. (a, b) Photograph of commercial PTFE waste strips. (c) SEM image the PTFE sample. (d, e) Reaction of PTFE pieces with NaK liquid alloy. (f) SEM image of the obtained PTFE-derived carbon.

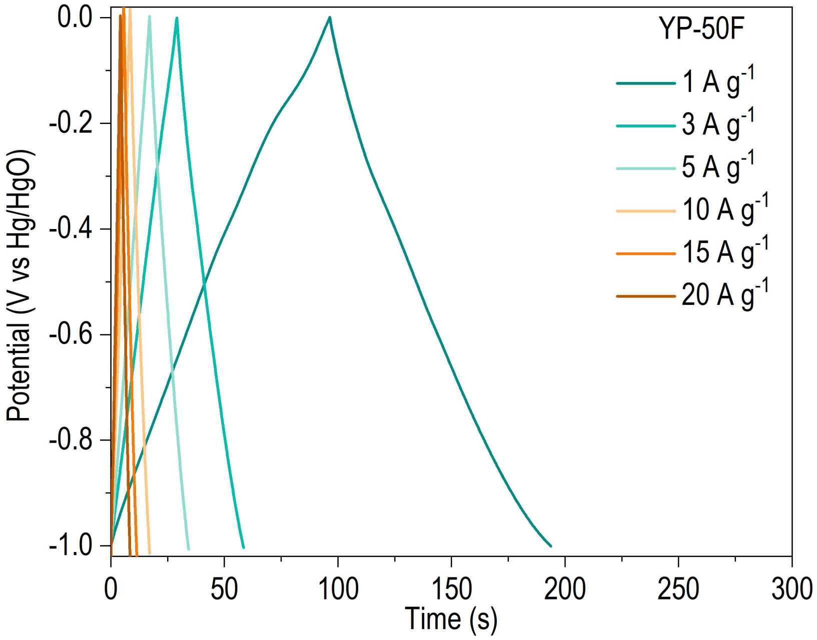

Fig. S15. Galvanostatic charge/discharge curves at 1-20 A g^{-1} for YP50F.

Supplementary Note 7: *Calculation of defluorination efficiency and fluoride yield of PTFE.*

To quantitatively evaluate the fluorine conversion during the CEC-SPR process, elemental analysis and ion chromatography were employed. After reaction, the solid products were quenched carefully with water, followed by filtration and vacuum drying. The black solid residue (PTFE-C) was subjected to elemental analysis, while the aqueous filtrate containing soluble fluoride species was analyzed by ion chromatography (**Table S1**).

Table S1. Elemental composition of pristine PTFE and the solid degradation residue, along with the calculated defluorination efficiency and yield.

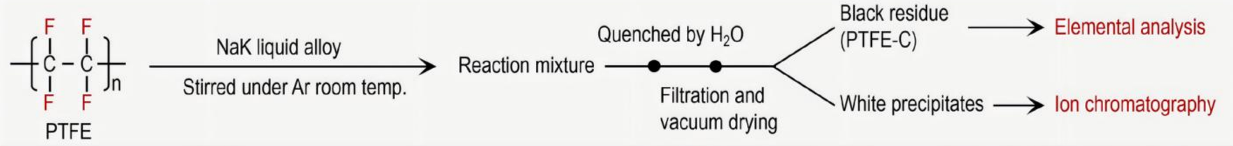

Entry	Sample	Anal. C(%)	H(%)	O(%)	N(%)	F(%)	Sum of C/H/O/N/F (%)	Others (%)	Ratio of F/C	F (%) lost from PTFE
1[a]	PTFE	24.02	-	-	-	75.98	100.00	-	3.164	-
2	Residue	56.39	3.63	15.45	4.90	15.95	96.32	3.68	0.282	91.1%

[a] Theoretical value calculated from the molecular formula of PTFE

The defluorination efficiency was calculated based on the fluorine-to-carbon mass ratio (F/C) of pristine PTFE and the degraded residue, assuming conservation of carbon during the degradation process. For pristine PTFE [$(C_2F_4)_n$], the theoretical F/C mass ratio is 3.164 (corresponding to 75.98 wt% F and 24.02 wt% C). The F/C ratio of the solid residue was determined from elemental analysis (F%/C%). The defluorination efficiency was then calculated as follows:

$$\text{Defluorination efficiency (\%)} = \left(1 - \frac{(F/C)_{residue}}{(F/C)_{PTFE}} \times 100\%\right)$$

The yield of NaF/KF was calculated as the molar ratio of fluoride ions recovered (measured by ion chromatography) to the theoretical fluorine content of the initial PTFE (75.98 wt%), assuming complete conversion of released F^- into NaF/KF:

$$\text{Yield (\%)} = \frac{nF^{-}, actual}{nF^{-}, theoretical}$$

Supplementary Note 8: *Calculation of defluorination efficiency of PFOA.*

In a typical recycling reaction, a mixture of PFOA (0.5 g) and NaK alloy (0.6135 g) was placed in a sealed stainless-steel reactor under an argon atmosphere. The reactor was then subjected to mechanical stimulation to initiate the contact-electro-catalysis induced self-propagating reaction (CEC-SPR). After the reaction was completed and the system cooled to room temperature, the solid residue was collected, thoroughly washed with deionized water several times, and dried under vacuum at 120°C for 12 h (**Fig. S16**). To quantitatively evaluate fluorine conversion during the CEC-SPR process, elemental analysis was performed on the resulting solid residue (**Table S2**).

Table S2. Elemental composition of pristine PFOA and the solid degradation residue, along with the calculated defluorination efficiency.

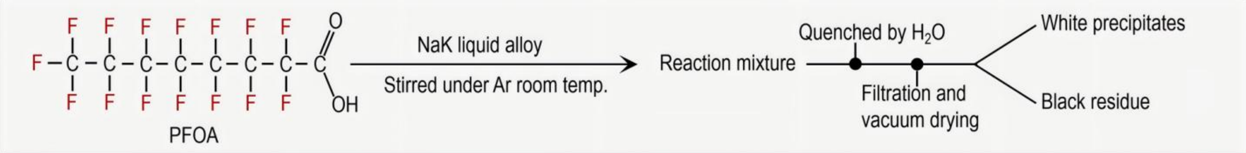

Entry	Sample	Anal. C(%)	H(%)	O(%)	N(%)	F(%)	Sum of C/H/O/N/F (%)	Others (%)	Ratio of F/C	F (%) lost from PFOA
1[a]	PFOA	23.20	0.24	7.73	-	68.83	100.00	-	2.966	-
2	Residue	79.18	5.88	8.40	0.17	4.70	98.33	1.67	0.059	98.0%

[a] Theoretical value calculated from the molecular formula of PFOA

The defluorination efficiency was calculated based on the fluorine-to-carbon mass ratio (F/C) of pristine PFOA and the degraded residue, assuming conservation of carbon during the degradation process. For pristine PFOA [$C_8HO_2F_{15}$], the theoretical F/C mass ratio is 2.966 (corresponding to 68.83 wt% F and 23.20 wt% C). The F/C ratio of the solid residue was determined from elemental analysis (F%/C%). The defluorination efficiency was then calculated as follows:

$$\text{Defluorination efficiency (\%)} = \left(1 - \frac{(F/C)_{residue}}{(F/C)_{PFOA}} \times 100\%\right)$$

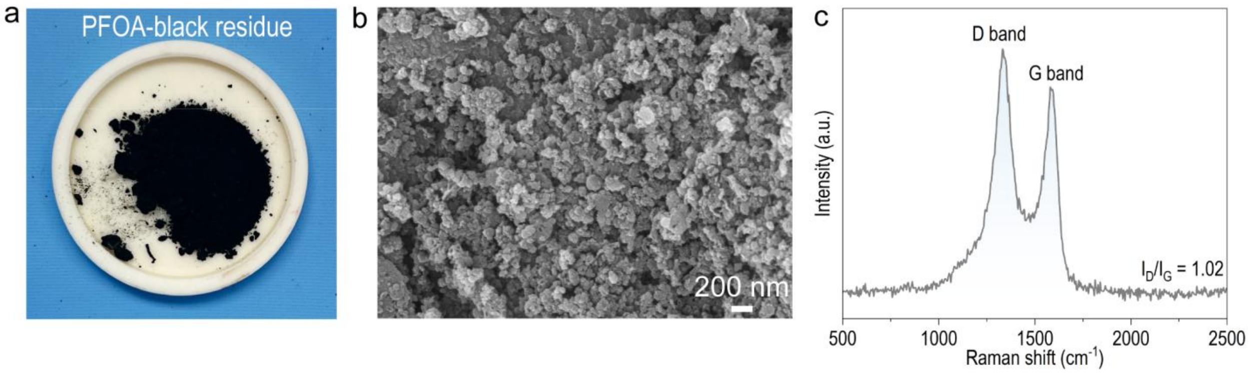

Fig. S16. Structural characterization of the PFOA-derived black residue. (a) Photograph, (b) SEM image, and (c) Raman spectrum of the PFOA-derived black residue.

Supplementary Note 9: *Calculation of defluorination efficiency of PFNA.*

In a typical recycling reaction, a mixture of PFNA (0.5 g) and NaK alloy (0.6155 g) was placed in a sealed stainless-steel reactor under an argon atmosphere. The reactor was then subjected to mechanical stimulation to initiate the contact-electro-catalysis induced self-propagating reaction (CEC-SPR). After the reaction was completed and the system cooled to room temperature, the solid residue was collected, thoroughly washed with deionized water several times, and dried under vacuum at 120°C for 12 h (**Fig. S17**). To quantitatively evaluate fluorine conversion during the CEC-SPR process, elemental analysis was performed on the resulting solid residue (**Table S3**).

Table S3. Elemental composition of pristine PFNA and the solid degradation residue, along with the calculated defluorination efficiency and yield.

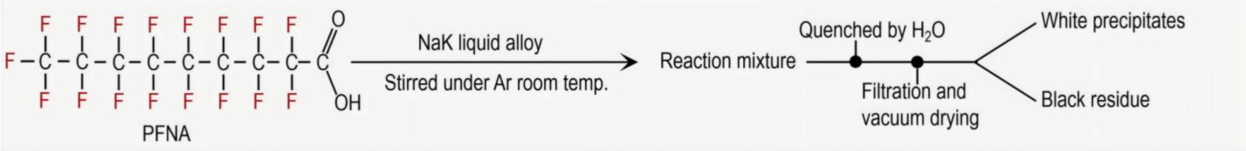

Entry	Sample	Anal. C(%)	H(%)	O(%)	N(%)	F(%)	Sum of C/H/O/N/F (%)	Others (%)	Ratio of F/C	F (%) lost from PFNA
1[a]	PFNA	23.29	0.22	6.89	-	69.60	100.00	-	2.988	-
2	Residue	71.00	1.95	13.89	0.33	11.86	99.03	0.97	0.167	94.4%

[a] Theoretical value calculated from the molecular formula of PFNA

The defluorination efficiency was calculated based on the fluorine-to-carbon mass ratio (F/C) of pristine PFNA and the degraded residue, assuming conservation of carbon during the degradation process. For pristine PFNA [$C_9HO_2F_{17}$], the theoretical F/C mass ratio is 2.988 (corresponding to 69.60 wt% F and 23.29 wt% C). The F/C ratio of the solid residue was determined from elemental analysis (F%/C%). The defluorination efficiency was then calculated as follows:

$$\text{Defluorination efficiency (\%)} = \left(1 - \frac{(F/C)_{residue}}{(F/C)_{PFNA}} \times 100\%\right)$$

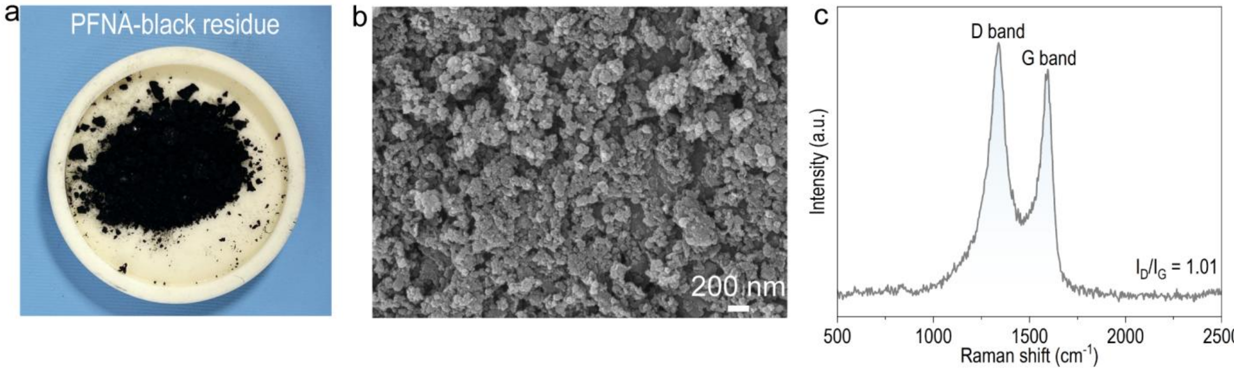

Fig. S17. Structural characterization of the PFNA-derived black residue. (a) Photograph, (b) SEM image, and (c) Raman spectrum of the PFNA-derived black residue.

Supplementary Note 10: *Calculation of defluorination efficiency of FEP.*

In a typical recycling reaction, a mixture of FEP (0.5 g) and NaK alloy (0.6775 g) was placed in a sealed stainless-steel reactor under an argon atmosphere. The reactor was then subjected to mechanical stimulation to initiate the contact-electro-catalysis induced self-propagating reaction (CEC-SPR). After the reaction was completed and the system cooled to room temperature, the solid residue was collected, thoroughly washed with deionized water several times, and dried under vacuum at 120°C for 12 h (**Fig. S18**). To quantitatively evaluate fluorine conversion during the CEC-SPR process, elemental analysis was performed on the resulting solid residue (**Table S4**).

Table S4. Elemental composition of pristine FEP and the solid degradation residue, along with the calculated defluorination efficiency and yield.

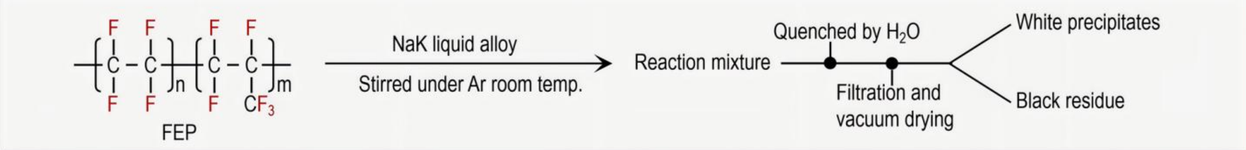

Entry	Sample	Anal. C(%)	H(%)	O(%)	N(%)	F(%)	Sum of C/H/O/N/F (%)	Others (%)	Ratio of F/C	F (%) lost from FEP
1[a]	FEP	25.00	-	-	-	75.00	100.00	-	3.000	-
2	Residue	57.83	2.35	19.10	0.33	18.27	97.88	2.12	0.316	94.7%

[a] Theoretical value calculated from the molecular formula of FEP

The defluorination efficiency was calculated based on the fluorine-to-carbon mass ratio (F/C) of pristine Fluorinated ethylene propylene (FEP) and the degraded residue, assuming conservation of carbon during the degradation process. For pristine FEP $[(C_2F_4)_n(C_3F_6)_m]$, assuming a fluorine content of 75 wt% (*i.e.*, 75.00 wt% F and 25.00 wt% C), the theoretical F/C mass ratio is 3.000. The F/C ratio of the solid residue was determined from elemental analysis (F%/C%). The defluorination efficiency was then calculated as follows:

$$\text{Defluorination efficiency (\%)} = \left(1 - \frac{(F/C)_{residue}}{(F/C)_{FEP}} \times 100\%\right)$$

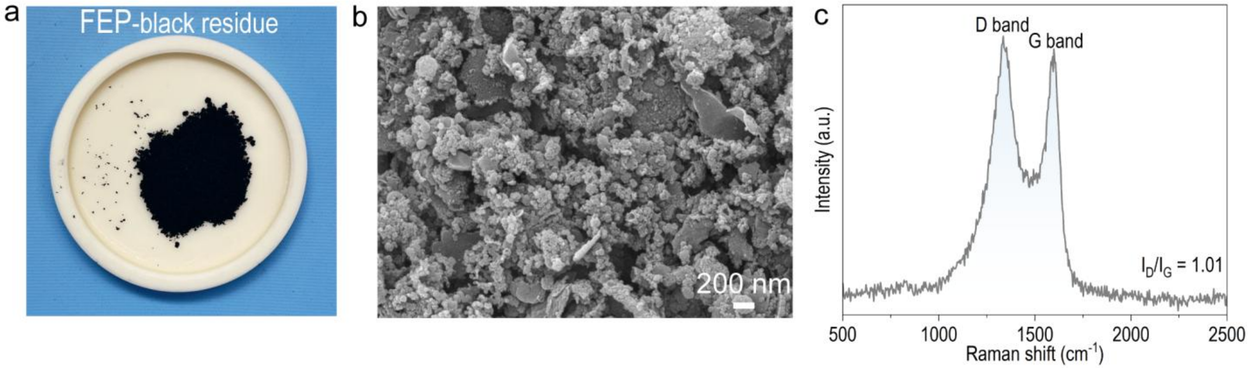

Fig. S18. Structural characterization of the FEP-derived black residue. (a) Photograph, (b) SEM image, and (c) Raman spectrum of the FEP-derived black residue.

Supplementary Note 11: *Calculation of defluorination efficiency of PFD.*

In a typical recycling reaction, a mixture of PFD (0.5 g) and NaK alloy (1.0165 g) was placed in a sealed stainless-steel reactor under an argon atmosphere. The reactor was then subjected to mechanical stimulation to initiate the contact-electro-catalysis induced self-propagating reaction (CEC-SPR). After the reaction was completed and the system cooled to room temperature, the solid residue was collected, thoroughly washed with deionized water several times, and dried under vacuum at 120°C for 12 h (**Fig. S19**). To quantitatively evaluate fluorine conversion during the CEC-SPR process, elemental analysis was performed on the resulting solid residue (**Table S5**).

Table S5. Elemental composition of pristine PFD and the solid degradation residue, along with the calculated defluorination efficiency and yield.

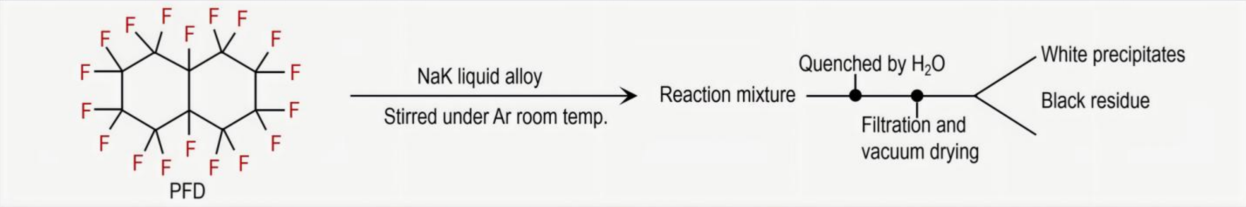

Entry	Sample	Anal. C(%)	H(%)	O(%)	N(%)	F(%)	Sum of C/H/O/N/F (%)	Others (%)	Ratio of F/C	F (%) lost from PFD
1[a]	PFD	26.00	-	-	-	74.00	100.00	-	2.848	-
2	Residue	72.00	2.37	9.71	0.51	13.06	97.65	2.35	0.181	93.6%

[a] Theoretical value calculated from the molecular formula of PFD

The defluorination efficiency was calculated based on the fluorine-to-carbon mass ratio (F/C) of pristine Perfluorodecalin (PFD) and the degraded residue, assuming conservation of carbon during the degradation process. For pristine PFD [$C_{10}F_{18}$], the theoretical F/C mass ratio is 2.848 (corresponding to 74.00 wt% F and 26.00 wt% C). The F/C ratio of the solid residue was determined from elemental analysis (F%/C%). The defluorination efficiency was then calculated as follows:

$$\text{Defluorination efficiency (\%)} = \left(1 - \frac{(F/C)_{residue}}{(F/C)_{PFD}} \times 100\%\right)$$

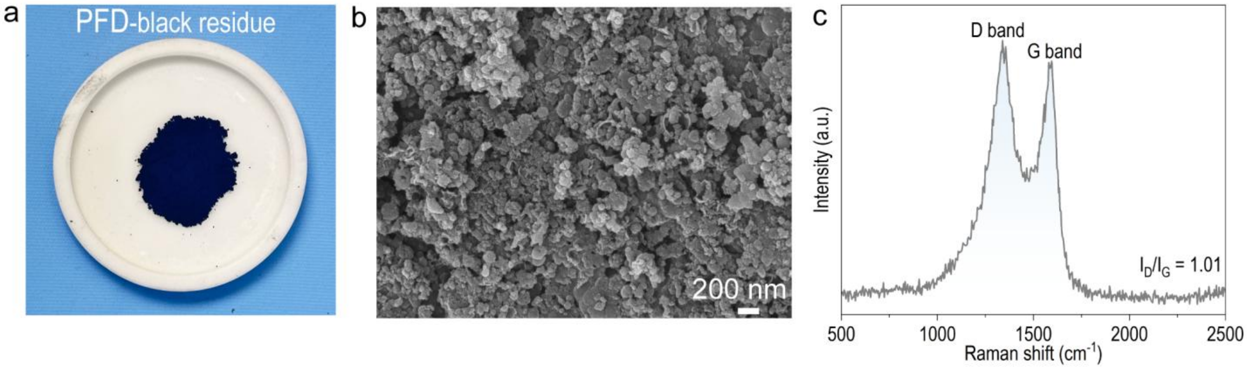

Fig. S19. Structural characterization of the PFD-derived black residue. (a) Photograph, (b) SEM image, and (c) Raman spectrum of the PFD-derived black residue.

Supplementary Note 12: *Calculation of defluorination efficiency of PFO.*

In a typical recycling reaction, a mixture of PFO (0.5 g) and NaK alloy (0.6960 g) was placed in a sealed stainless-steel reactor under an argon atmosphere. The reactor was then subjected to mechanical stimulation to initiate the contact-electro-catalysis induced self-propagating reaction (CEC-SPR). After the reaction was completed and the system cooled to room temperature, the solid residue was collected, thoroughly washed with deionized water several times, and dried under vacuum at 120°C for 12 h (**Fig. S20**). To quantitatively evaluate fluorine conversion during the CEC-SPR process, elemental analysis was performed on the resulting solid residue (**Table S6**).

Table S6. Elemental composition of pristine PFO and the solid degradation residue, along with the calculated defluorination efficiency and yield.

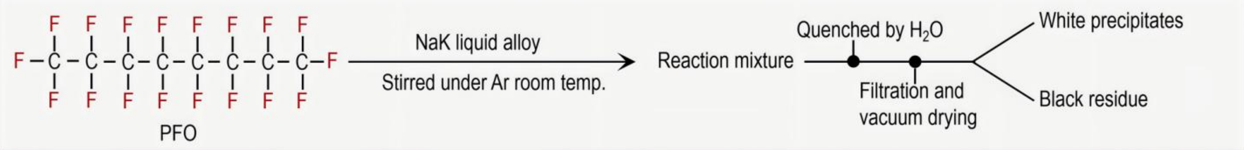

Entry	Sample	Anal. C(%)	H(%)	O(%)	N(%)	F(%)	Sum of C/H/O/N/F (%)	Others (%)	Ratio of F/C	F (%) lost from PFO
1[a]	PFO	21.93	-	-	-	78.07	100.00	-	3.559	-
2	Residue	70.30	2.35	12.20	0.33	13.33	98.51	1.49	0.190	94.7%

[a] Theoretical value calculated from the molecular formula of PFO

The defluorination efficiency was calculated based on the fluorine-to-carbon mass ratio (F/C) of pristine perfluorooctane (PFO) and the degraded residue, assuming conservation of carbon during the degradation process. For pristine PFO [C_8F_{18}], the theoretical F/C mass ratio is 3.559 (corresponding to 78.07 wt% F and 21.93 wt% C). The F/C ratio of the solid residue was determined from elemental analysis (F%/C%). The defluorination efficiency was then calculated as follows:

$$\text{Defluorination efficiency (\%)} = \left(1 - \frac{(F/C)_{residue}}{(F/C)_{PFO}} \times 100\%\right)$$

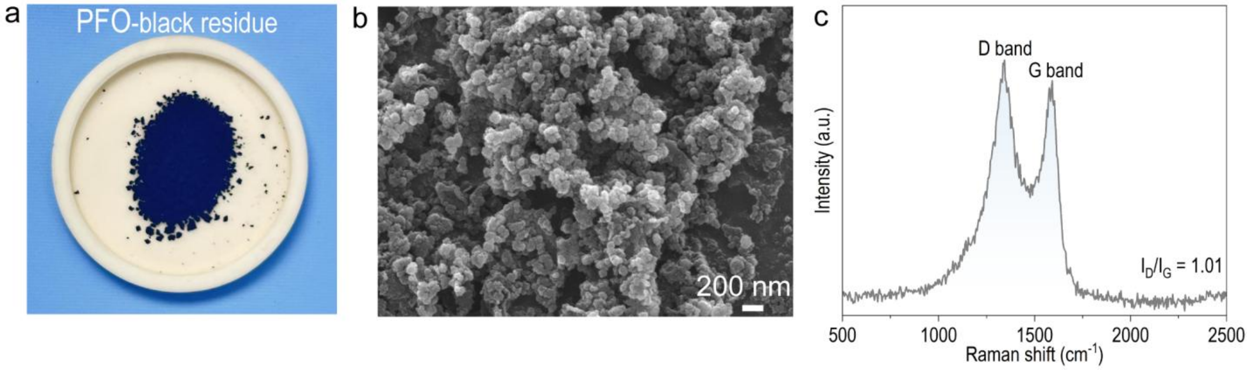

Fig. S20. Structural characterization of the PFO-derived black residue. (a) Photograph, (b) SEM image, and (c) Raman spectrum of the PFO-derived black residue.
